# Derivational Morphology in Agrammatic Aphasia: A Comparison Between Prefixed and Suffixed Words

**DOI:** 10.3389/fpsyg.2020.01070

**Published:** 2020-05-29

**Authors:** Laura Anna Ciaccio, Frank Burchert, Carlo Semenza

**Affiliations:** ^1^Potsdam Research Institute for Multilingualism, University of Potsdam, Potsdam, Germany; ^2^Linguistics Department, University of Potsdam, Potsdam, Germany; ^3^Department of Neuroscience (Padua Neuroscience Center), University of Padua, Padua, Italy; ^4^IRCCS San Camillo Hospital Foundation, Neuropsychology Unit, Lido-Venice, Italy

**Keywords:** Broca’s aphasia, morphological decomposition, morphological errors, derivation, prefixes

## Abstract

Although a relatively large number of studies on acquired language impairments have tested the case of derivational morphology, none of these have specifically investigated whether there are differences in how prefixed and suffixed derived words are impaired. Based on linguistic and psycholinguistic considerations on prefixed and suffixed derived words, differences in how these two types of derivations are processed, and consequently impaired, are predicted. In the present study, we investigated the errors produced in reading aloud simple, prefixed, and suffixed words by three German individuals with agrammatic aphasia (NN, LG, SA). We found that, while NN and LG produced similar numbers of errors with prefixed and suffixed words, SA showed a selective impairment for prefixed words. Furthermore, NN and SA produced more errors specifically involving the affix with prefixed words than with suffixed words. We discuss our findings in terms of relative position of stem and affix in prefixed and suffixed words, as well as in terms of specific properties of prefixes and suffixes.

## Introduction

A series of studies on acquired language impairments have focused on linguistic morphology, i.e., the domain of linguistics that is concerned with how complex words, such as compounds (e.g., *paycheck*), derived words (*payment*), and inflected words (*pays*), are formed and internally structured. These studies involved individuals whose comprehension or production of complex words, as compared to simple words, is impaired. This condition is referred to as “morphological impairment” and it has mainly been reported in individuals with agrammatic aphasia, generally of Broca’s type, as well as in individuals with deep or phonological dyslexia (e.g., Luzzatti et al., 2001; Rastle et al., 2006; Semenza and Mondini, 2015). Impairments of morphology have also been reported in cases of fluent aphasia (“jargon aphasia”: Caplan et al., 1972; Semenza et al., 1990), as well as in other neuropsychological disorders, such as semantic dementia (Auclair-Ouellet et al., 2016a, b) or neglect (Semenza et al., 2011; Marelli et al., 2013; Reznick and Friedmann, 2015). In production, morphological impairments are characterized by so-called “morphological” or “constituent” errors, i.e., errors that affect one of the constituent morphemes, while sparing the other. Examples are morpheme substitutions and omissions, affecting stems (e.g., ≪baseball≫ or ≪ball≫ instead of *volleyball*) or affixes (≪player≫ or ≪play≫ instead of *playful*), and semantic paraphasias (e.g., ≪without contact≫ instead of *contactless*). The label “morphological error” is generally found in studies focusing on derivation or inflection, referring to errors that affect affixes while sparing stems. Instead, the label “constituent error” is more often found in studies that focus on compounds, to describe errors involving one of the stems. Morphologically based errors have been taken as evidence that complex words are accessed and represented in a decomposed fashion (e.g., *playful* [play][-ful]), i.e., based on the single constituents of which they are composed (Semenza and Mondini, 2015).

Studies investigating the impairment of derived words have mostly focused on the case of suffixed forms, e.g., words such as *payer* in which the affix follows the stem (e.g., Rastle et al., 2006). Instead, prefixed words such as *prepay*, in which the affix is placed before the stem, have been comparably neglected. A study specifically focusing on derivation by prefixation is the one by Semenza et al. (2002). The authors tested two speakers of Slovenian with non-fluent aphasia in reading aloud, repetition, and writing to dictation tasks. The two participants produced large number of errors which preserved the prefix while affecting the stem, while cases in which the prefix was substituted or omitted were comparably less frequent. This is an unusual pattern for morphological errors with derived words, at least with suffixed words, for which it is generally the stem that is preserved while the affix is affected (Kay, 1988; Miceli and Caramazza, 1988; Rastle et al., 2006). Unfortunately, because the study did not include suffixed words, we do not know whether their preservation of affixes was a general characteristic of their impairment or, on the contrary, this pattern was restricted to prefixed words. Furthermore, all the prefixes included in the study were at the same time existing Slovenian prepositions, and hence free morphemes, which possibly makes the stimuli more similar to compounds. Other studies have tested sets of prefixed words in addition to sets of suffixed words (e.g., Job and Sartori, 1984; Kay, 1988; Hamilton and Coslett, 2008). These have all reported impaired use of prefixation in addition to impaired suffixation. Yet, none of them has specifically compared prefixed to suffixed words to investigate whether there are differences in how they are impaired.

The lack of studies on acquired morphological disorders directly comparing prefixed and suffixed words is particularly striking if we consider that differences between these two types of derived words have been described both in the linguistics and in the psycholinguistics literature. From a linguistic point of view, prefixes and suffixes have been claimed to serve different functions. This claim is mostly based on the notion of “head”, i.e., the constituent that determines the grammatical properties (such as gender or word class) of the complex word. While, in suffixed words, it is generally the suffix that functions as head, the head of prefixed words is generally their stem, as predicted by Williams’ (1981) “Right-Hand Head Rule”, according to which the head tends to be the most right-hand constituent (but note that there are some exceptions, since prefixes can sometimes be heads, e.g., *en-* in *encourage*). Based on this difference between prefixes and suffixes, some linguists have claimed that derivation by prefixation and derivation by suffixation should be classified as two distinct types of word-formation processes (e.g., Kastovsky, 2005). When it comes to the typological distribution of prefixing and suffixing morphology in the world’s languages, a universal preference for suffixation has been observed (Greenberg, 1963). Cutler et al. (1985) have explained this preference in terms of the cognitive mechanisms underlying language processing: processing complex words is easier when the lexical-semantic information carried by the stem comes in the first, and most salient, portion of the word, followed by the affix, which rather serves the processing of larger syntactic and semantic units.

Psycholinguistic evidence on the processing of prefixed derived words is mixed. A number of visual word recognition studies using the masked priming technique have reported significant priming effects for prefixed words (e.g., Diependaele et al., 2009; Kazanina, 2011; Ciaccio and Clahsen, 2020), i.e., shorter lexical decision times to target words when these are preceded by a prefixed prime (e.g., *prepay-pay*) as compared to a baseline condition (unrelated prime: *precook-pay*). Morphological priming effects have been taken as a diagnostic of efficient morphological decomposition of the prime into affix and stem (e.g., [pre-][pay]), which leads to pre-activation of the target (*pay*) before it is actually presented (Marslen-Wilson, 2007). However, there is also evidence that stem access or, more in general, morphological decomposition may be delayed or more costly in prefixed words as compared to suffixed words. In lexical decision tasks, Ferrari Bridgers and Kacinik (2017) found that prefixed words elicit longer response latencies than suffixed words. Similarly, Bergman et al. (1988) reported that, while suffixed and pseudo-suffixed words have similar recognition times, suggestive of automatic stem access, pseudo-prefixed words are recognized more slowly than prefixed words. Finally, Colé et al. (1989) found cumulative root frequency effects on lexical decision times in the case of suffixed words, but not in prefixed words, while Beauvillain (1996) found effects of root frequency on first fixation durations for suffixed words, but only in later measures such as second fixation durations for prefixed words. All these findings have been explained in terms of more effortful morphological decomposition of prefixed words as compared to suffixed words. Prefixes and suffixes may also be processed differently because of the information they encode, with prefixes mostly encoding semantic information, and suffixes additionally carrying a grammatical function. For example, Beyersmann et al. (2015) found that, in a letter identification task, reaction times were longer when the target letter was embedded in a suffix than in a non-morphological ending, while response times for letters contained in prefixes and pseudo-prefixes did not differ. The authors concluded that suffixes, but not prefixes, are identified as sub-lexical chunks during reading, which they explained in terms of the different functions of prefixes and suffixes.

Investigating how prefixed and suffixed words are impaired in acquired morphological disorders can help better understand whether there are differences in how they are processed. Indeed, previous research on morphological impairments have highlighted differences between different types of complex words that would not have been detected in some psycholinguistic tasks, especially in the most widespread task in morphological processing research, namely masked priming. For example, differences between derived and inflected words have been reported in acquired language impairments (e.g., Tyler and Cobb, 1987; Miceli and Caramazza, 1988), although masked priming effects with derived and inflected primes have been consistently shown to be similar in magnitude, at least in adult native speakers (e.g., Jacob et al., 2018). Similarly, while the distinction between compound head and modifier does not modulate masked morphological priming effects (e.g., Duñabeitia et al., 2009; Beyersmann et al., 2018), this has been shown to play a role for morphological impairments, in which head constituents are retained better than modifiers (Jarema et al., 2010; Semenza et al., 2011; Marelli et al., 2013). An additional advantage of investigating morphologically complex words in acquired language impairments is that it is possible to obtain data from naturalistic tasks, such as reading aloud, without needing sophisticated and relatively artificial experimental settings.

## The Present Study

A relatively large number of studies have investigated dissociations in the impairment of different types of complex words in acquired morphological disorders (e.g., Tyler and Cobb, 1987; Miceli and Caramazza, 1988; Luzzatti et al., 2001; Penke and Krause, 2002; Hamilton and Coslett, 2008; Marusch et al., 2012). However, none of these have specifically focused on the distinction between derivation by prefixation and by suffixation. As a consequence, we do not know if and to what extent morphological impairments affect all derived words in the same way or, on the contrary, there are differences in how prefixed and suffixed words are impaired. Therefore, the present study specifically investigated errors in reading aloud prefixed and suffixed words in subjects with acquired morphological impairments. We did so by taking advantage of the rich derivational morphology of German, which, compared to other Indo-European languages, has a larger inventory of derivational prefixes (see e.g., Smolka et al., 2014; Günther et al., 2019).

We recruited German individuals with agrammatic aphasia, which is characterized by impaired use of grammatical materials such as function words and inflected forms (Goodglass and Menn, 1985) as well as by impaired production of morphologically complex words (Semenza and Mondini, 2015). Speakers with agrammatic aphasia have been shown to vary consistently in the numbers and types of errors that they produce with complex words (Miceli et al., 1989). The present study extends the current evidence by investigating whether and how errors vary systematically for prefixed and suffixed derived words, and if such error patterns can be predicted based on the differences between prefixed and suffixed words that have been described in the linguistics and psycholinguistics literature.

The question whether prefixed and suffixed words are impaired in the same way is not trivial. On the one hand, we might expect that the different properties of prefixed and suffixed words have consequences on how these are impaired. On the other, it is also possible that morphological impairments equally affect all derivational phenomena, i.e., all words in which a derivational affix is added to a stem, irrespective of the order in which these two constituents occur or of the different properties of prefixes and suffixes. Based on previous psycholinguistic evidence for differences between prefixed and suffixed words in language-unimpaired populations, we regarded the former hypothesis as more promising than the latter.

In the present study, we investigated the numbers and types of errors produced in reading aloud prefixed and suffixed words. We first asked the question whether individuals with morphological impairments produce more errors with, respectively, prefixed and suffixed words as compared to simple words. In line with the typical profile of a morphological impairment (e.g., Job and Sartori, 1984; Rastle et al., 2006; Lorenz and Zwitserlood, 2014), we expected both prefixed and suffixed words to yield more errors than simple words. However, if it is true that processing prefixed words is more costly than processing suffixed words because of the word-final position of the stem (Bergman et al., 1988; Colé et al., 1989; Beauvillain, 1996; Ferrari Bridgers and Kacinik, 2017), then prefixed words may be more impaired than suffixed words. We then focused on the types of errors produced, and specifically on the likelihood of producing an error specifically affecting the prefix or the suffix. Because suffixes and prefixes both contribute to the meaning of the derived word but suffixes also have a more prominent grammatical function, prefixes may be more prone to be lost than suffixes. This should lead to a larger number of affix errors (possibly omissions), with prefixes compared to suffixes. Finally, if stems in prefixed words are less accessible than stems in suffixed words, these may also be less retained, leading to more errors on stems in prefixed than in suffixed words.

## Materials and Methods

### Participants

Three German individuals (NN, LG, SA) participated in the study. They were recruited through the database of persons with aphasia (PwAs) of the Linguistics Department of the University of Potsdam, based on their diagnosis for aphasia of Broca’s type. Biographic information as well as details about the participants’ language impairment are provided in Table 1. All PwAs’ spontaneous speech was characterized by the typical symptoms of agrammatic speech: simplified, incomplete or ungrammatical sentences, mostly main clauses with no or few subordinate clauses, often missing verbs or verbal inflection. All PwAs were well oriented in space and time and had normal or corrected-to-normal vision. None of them suffered from visual neglect. They were all informed about the aims and contents of the study and signed a written consent form.

**TABLE 1:** PwAs’ biographic information.

**Information**	**NN**	**LG**	**SA**
Gender	M	M	F
Age	63	75	50
Education	university degree	vocational training	vocational training
Handedness	left	right	right
Lesion hemisphere	right	left	left
Time post onset	22; 4	19; 11	15; NA
Speech output	agrammatic	agrammatic	agrammatic
Other impairment(s)	mild dysarthria	−	−

The PwAs were previously assessed by means of the Aachen Aphasia Test (*Aachener Aphasie Test*; AAT; Huber et al., 1983). Results from the sub-tests on repetition, written language, naming, and comprehension are provided in Table 2. All participants globally showed a profile of mild-to-medium aphasia, though with some differences between individuals and tasks. NN resulted only mildly impaired in language comprehension, and considerably less impaired than both LG and SA. In written language tasks, NN’s impairment resulted to be medium-to-mild, while, again, LG’s and SA’s performance was worse, pointing to a mild-to-severe impairment. In repetition, the three PwAs had similar performance, suggesting a medium level of impairment.

**TABLE 2:** Summary of PwAs’ performance in the Aachen Aphasia Test.

**Task**	**NN**	**LG**	**SA**
**Repetition**	**50**	**46**	**44**
Sounds	28 (93%)	25 (83%)	29 (97%)
Monosyllabic words	25 (83%)	25 (83%)	29 (97%)
Foreign words	27 (90%)	22 (73%)	28 (93%)
Complex words	19 (63%)	20 (67%)	15 (50%)
Sentences	12 (40%)	13 (43%)	3 (10%)
**Written Language**	**66**	**34**	**39**
Reading aloud	26 (87%)	15 (50%)	22 (73%)
Composite to dictation	22 (73%)	12 (40%)	7 (23%)
Write to dictation	17 (57%)	NA	4 (13%)
**Naming**	**57**	**62**	**43**
Objects: simple words	24 (80%)	26 (87%)	21 (70%)
Colors: adjectives	23 (77%)	24 (80%)	21 (70%)
Objects: compound nouns	21 (70%)	22 (73%)	15 (50%)
Situations and actions	17 (57%)	17 (57%)	11 (37%)
**Comprehension**	**83**	**56**	**59**
Auditory word comprehension	30 (100%)	30 (100%)	20 (67%)
Auditory sentence comprehension	20 (67%)	20 (67%)	22 (73%)
Word reading comprehension	29 (97%)	20 (67%)	22 (73%)
Sentence reading comprehension	20 (67%)	14 (47%)	21 (70%)

*Results of the AAT subcomponents in bold are provided in terms of percentile ranks. Percentile ranks over 90 indicate no aphasia, ranks between 90 and 60 indicate mild aphasia, between 60 and 30 medium aphasia, and below 30 severe aphasia. Results of the single tasks are provided in terms of number of points obtained. In each task, a maximum of 30 points can be obtained; three points are assigned to correct responses and 0 points to incorrect responses, while 2 or 1 points can be assigned for intermediate cases, e.g., if the produced word or sentence had some degree of similarity to the stimulus.*

We additionally designed an ad-hoc test to assess PwAs’ performance with complex words, testing reading aloud, repetition, visual lexical decision, and auditory lexical decision. Items in the reading aloud and repetition tasks were matched for morphological complexity, length, and frequency; so were the items in the two lexical decision tasks (visual and auditory). Table 3 presents details about the materials of the test and its outcome. Overall, although the PwAs varied in the number of errors they produced, reading aloud complex words was severely impaired in all of them, and significantly more impaired than repetition. Considering that, for all PwAs, performance in the visual lexical decision task was comparably better, their impairment does not seem to be located in the morphological processes that are specifically tied to orthographic processing in the input modality^1^. A qualitative analysis of the errors produced in reading aloud revealed that four out of 13 errors (30.8%) made by NN were substitutions or omissions of affixes (e.g., *Vertreter* “representative”: ≪vertreten≫ “represent”), one error was an insertion of an ending between two morphemes (*freundschaftlich* “friendly”: ≪^∗^freundeschaftlich≫ ^∗^friendsly), one error was possibly visual (*decken* “cover”: ≪stecken≫ “insert”), one was a substitution with an unrelated word, and six were word fragments, omissions, or non-meaningful letter strings. In the case of LG, ten of 17 errors (58.8%) were affix substitutions, six were word fragments, omissions, or non-meaningful letter strings, and one was a substitution that may be classified as visual error. As for SA, of her 18 errors, nine (50%) were affix substitutions or omissions, one was a visual error, two were substitutions with unrelated words, and six were omissions or non-meaningful letter strings.

**TABLE 3:** Mean item properties (standard deviation in parenthesis) and summary of PwAs’ performance in the ad-hoc preliminary test.

	**Item properties**		**Number of correct responses**		
**Task**	**Length (letters)**	**Zipf frequency**	**NN**	**LG**	**SA**
Reading aloud (N = 20)	9.85 (2.72)	3.28 (0.83)	7 (35%)	3 (15%)	2 (10%)
Repetition (N = 20)	9.75 (2.71)	3.27 (1.11)	16 (80%)	11 (55%)	12 (60%)
Reading aloud vs repetition			χ^2^ = 8.286,	χ^2^ = 7.033,	χ^2^ = 10.989,
			*p* = 0.0040*	*p* = 0.0080*	*p* = 0.0009*
Visual lexical decision (N = 35)			26 (74%)	25 (71%)	26 (74%)
*Existing words (N = 25)*	9.32 (2.29)	3.06 (1.20)	19 (76%)	18 (72%)	20 (80%)
*Non-words (N = 10)*	9.40 (2.46)	−	7 (70%)	7 (70%)	6 (60%)
Auditory lexical decision (*N* = 35)			28 (80%)	25 (71%)	24 (69%)
*Existing words (N = 25)*	9.36 (2.48)	3.20 (1.21)	19 (76%)	18 (72%)	17 (68%)
*Non-words (N = 10)*	9.60 (2.67)	−	9 (90%)	7 (70%)	7 (70%)
Visual vs auditory lexical decision			χ^2^ = 0.324,	χ^2^ = 0,	χ^2^ = 0.280,
			*p* = 0.5692	*p* = 1	*p* = 0.5967

*Item types in the reading aloud and repetition tasks: four compounds (two inflected), three prefixed derivations (one inflected), four suffixed derivations (all inflected), two derivations with two affixes (two suffixes or prefix and suffix), three inflected words, four simple words (2–3 syllables). Item types in the visual and auditory lexical decision task: 25 existing words, of which six compounds (two inflected), three suffixed derivations (all inflected), two prefixed derivations (one inflected), three derivations with two affixes (prefix + suffix), nine inflected words (two irregular), two simple words (3–4 syllables); 10 non-existing words, of which eight items created from existing words, deleting, adding, or substituting 1–2 letters from two simple words, one prefixed word, one suffixed word, two compound words, two inflected words; one illegal combination of stem with prefix; one illegal combination of stem with two suffixes.*

A control group of eight participants without language or neurological impairments additionally took part in the main experimental task. The control group was comparable for age (mean 67.4, SD 3.8, range 62–70), gender (4 men, 4 women), and education background (four with university degree, four with vocational training education) to the three PwAs.

### Materials

The experimental items were 60 simple words, 60 prefixed words, and 60 suffixed words. Each condition contained 20 adjectives, 20 nouns, and 20 verbs. All derived words consisted of one stem and one derivational morpheme; all verbs additionally contained the inflectional affix *-en*, which is the infinitival ending. Item characteristics are presented in Table 4. Following Sassenhagen and Alday (2016), matching information of the items across conditions is presented in terms of descriptive (mean, SD, range) rather than inferential statistics. Because suffixes tended to be longer than prefixes, we matched the items in the prefixed and suffixed condition for the length of their stems. All items were selected from a list of words that had been previously rated by German native speakers for imageability and semantic transparency, on a 1–7 scale (1 = lowest imageability/transparency), in two online surveys. Twenty-three subjects (19 women; mean age 33.96, SD 14.79) participated in the imageability survey, which included 369 simple and complex words, and twenty subjects (15 women; mean age 33.35, SD 9.97) participated in the transparency survey, which contained 321 complex words. Number of neighbors (Coltheart’s count, absolute), as well as word-form and lemma frequency were extracted from the dlex database (Heister et al., 2011). We additionally computed affix frequency by extracting the number of lemmas beginning with the letter string corresponding to each prefix, as well as the number of lemmas ending with the letter string corresponding to each suffix, normalized by the number of types included in the corpus. Frequency is provided in zipf-scale (Heuven et al., 2014). Items in all conditions and, if applicable, their stems and affixes had similar frequency distributions (in both word-form and lemma frequency) as well as similar distributions in terms of number of neighbors, imageability, and transparency. Finally, items in the different conditions were also similar in terms of phonological complexity. Respectively, 22 simple items (36.7%), 24 prefixed items (40%), and 16 suffixed items (26.7%) contained complex onsets, while 11 simple items (18.3%), 23 prefixed items (38.3%), and 21 suffixed items (35%) contained complex codas. Only three items in each condition contained hiatuses.

**TABLE 4:** Stimuli properties (mean, SD, range) of the experimental task.

	**Suffixed (*N* = 60)**	**Prefixed (*N* = 60)**	**Simple (*N* = 60)**
**Property**			
Transparency	6.05 (0.64)	5.64 (1.01)	
	3.56–6.76	3.15–6.86	
Imageability	3.97 (1.26)	3.83 (1.14)	4.43 (1.23)
	2.00–6.52	2.04–6.33	2.26–6.52
Affix frequency	6.39 (0.42)	6.65 (0.40)	
	5.84–7.67	5.60–7.07	
Length (letters)	9.00 (1.73)	8.32 (1.57)	7.23 (1.16)
	6–13	6–12	6–10
Stem length (letters)	5.62 (1.37)	5.60 (1.33)	
	3–9	3–9	
Word-form frequency	3.17 (0.58)	2.94 (0.65)	3.45 (0.53)
	1.69–4.71	1.21–4.71	1.51–4.23
Stem word-form frequency	4.37 (0.64)	4.38 (0.61)	
	2.98–5.60	2.92–5.37	
Lemma frequency	3.48 (0.52)	3.31 (0.63)	3.90 (0.44)
	2.31–4.79	1.61–4.78	2.48–4.69
Stem lemma frequency	4.61 (0.61)	4.78 (0.60)	
	2.99–5.83	3.53–5.85	
Number of neighbors	2.27 (2.17)	1.95 (1.31)	6.43 (5.34)
	0–9	0–5	0–25
Stem number of neighbors	18.15 (16.21)	13.85 (10.17)	
	0–76	1–44	

All materials were tested twice, in two separate sessions, with an interval of at least one week between the two sessions, so that, in total, each subject read 120 simple words, 120 prefixed words, and 120 suffixed words. Complete lists of the experimental items and of the affixes used in the experiment are provided in the Appendix Tables A1, A2, A3, and A4.

### Procedure

The experimental sessions took place at the participants’ homes, under quiet conditions. The experiment was run on a Macintosh Air 13^″^, using the software PsychoPy (Peirce, 2007), version 1.82.00. Each stimulus was presented in isolation, in lowercase letters (the first letter being capitalized in the case of nouns, as by default in German), in the middle of a computer screen. Participants were instructed to read the word silently and press the space bar when they had finished reading the word. Immediately after pressing the space bar or after a timeout (7 s), a countdown automatically appeared on the screen for 5 s. This was followed by a production cue (an exclamation mark). When the production cue appeared, participants were expected to produce the word that they had just read. A maximum of 4 s were available for each individual response, after which the next target word appeared automatically. Delayed reading was preferred over immediate reading to ensure that speakers had enough time to pre-process the target word and to reduce effects of item properties (e.g., Ferrand, 2000; Sulpizio et al., 2015). All responses were recorded using an external microphone and automatically stored locally by the experimental software. A total of 240 items were presented for reading aloud, 180 of which were experimental items and 60 were fillers. All items were distributed over four blocks. Within each block, items were presented in a randomized order. Each block was followed by a break. All participants saw the four blocks in a different order and they were tested with the reversed order of blocks in the second experimental session.

The study was approved by the ethics committee of the University of Potsdam (application number 32/2016). All participants received remuneration for their participation in the study. All participants signed an informed consent prior to their participation, in accordance with the Declaration of Helsinki.

### Data Analysis

We performed separate analyses for each of the three PwAs. For all analyses, we used binomial logistic regression models, as computed with generalized linear mixed effect models using the package lme4 (Bates et al., 2015) in the software R (version 3.6.2; R Core Team, 2018).

For the analysis testing the effect of Condition on error rates, responses were classified as “error” or “correct,” respectively, coded with 1 and 0 for the logistic regressions. Condition had three levels: simple, prefixed, and suffixed; “simple” was set as baseline, so that the models compared the performance with, respectively, prefixed and suffixed words to performance with simple words. We then conducted analyses on the types of errors produced with prefixed and suffixed words by each PwA, specifically focusing on affix errors and errors on stems. In line with previous literature (e.g., Rastle et al., 2006), we classified as “affix error” any error in which the stem was preserved while the affix was not produced correctly, i.e., it was either omitted (*unsauber* [un-][sauber] “not clean”: ≪sauber≫ “clean”, NN), substituted (*machtlos* [macht][-los] “powerless”: ≪machtvoll≫ [macht][-voll] “powerful”, SA), or substituted with non-lexical letter strings (neologisms; *erdenken* “think up” [er-][denken]: ≪*^∗^*kaldenken≫, NN). Errors on stems were errors in which the affix was preserved but the stem was either substituted with another existing stem (e.g., *drahtlos* [draht][-los] “wireless”: ≪gnadenlos≫ [gnaden][-los] “merciless”, SA) or with a neologism, which was generally phonologically similar to the stem (e.g., *unreif* [un-][reif] “immature”: ≪^∗^ungleif≫, LG). For each PwA, we fitted two binomial logistic regression models having as dependent variable, respectively, the occurrence of affix errors and the occurrence of errors on stems, coded with 1, as compared to any other output, coded with 0 (see Marelli et al., 2013). All models included Condition as fixed effect, with two levels (prefixed, suffixed; baseline = prefixed).

Other error types included whole-word substitutions with other complex words (e.g., *Urpflanze* [ur-][pflanze] “primordial plant”: ≪Unwetter≫ [un-][wetter] “storm”, NN), with simple words (e.g., *kraftlos* [kraft][-los] “powerless”: ≪frei≫ “free”, SA), or with neologisms (e.g., *unklar* [un-][klar] “unclear”: ≪^∗^urklei≫, NN), whole-word omissions, affix insertions (e.g., *unwichtig* [un-][wichtig] “unimportant” [un-][wichtig][-keit]: ≪Unwichtigkeit≫ “unimportance”, LG; *Pflanze* “plant”: ≪Pflanzen≫ [Pflanze][-n] “plants”, NN), and letter deletions in the -*en* infinitival verb endings (e.g., *leugnen* “ (to) deny”: ≪leugne≫ “(I) deny”; note the three conditions contained the same number of verbs). Immediate repairs were scored as correct, while long hesitations, interruptions after a word fragment, and null reactions were scored as whole-word omissions. With the main goal of not confounding articulatory difficulty with reading errors (especially in the case of NN, who had mild dysarthria), phonological errors were counted as correct responses if the distortion or insertion only involved one phoneme, thus allowing the stem or the affix to be clearly recognizable (see Marusch et al., 2012; Marusch et al., 2017). The few cases in which participants were disturbed by external factors or refused to complete the task due to tiredness were not included in the total count of items presented.

All models included random intercepts for items. Binomial logistic regression models allowed for additionally testing for inclusion of the following covariates: Session, to account for the fact that all items were tested twice; Trial Number, to account for training or fatigue effects throughout the experiment; and some relevant psycholinguistic variables (transparency, imageability, stem length, word-form and lemma frequency of full form and stem, affix frequency, neighbors of full form and stem). This way, any significant effect of Condition is controlled for (i.e., goes beyond) any effect of item characteristics or artifacts from the experimental setup, such as trial number and session (Sassenhagen and Alday, 2016). We first tested for inclusion of each covariate separately, and then of the relevant random slopes by items for all fixed factors. Covariates and random slopes were only added if they significantly improved the model fit, which we tested by comparing the simpler model to the more complex model via likelihood ratio chi-square tests (Baayen, 2008). For some of the models we fitted, the covariates did have a significant effect, while including random slopes never improved the model fit for any of the analyses. In the Results section, we report results concerning our main predictor (Condition) and the covariates that significantly improved the fit of the models.

## Results

The language-unimpaired subjects from the control group produced exclusively correct responses, except for one subject who omitted two prefixed items. Error rates and types of the three PwAs are summarized in Table 5.

**TABLE 5:** Summary of PwAs’ error rates and types in the experimental task.

**Errors**	**NN**			**LG**			**SA**		
	**Simple**	**Prefixed**	**Suffixed**	**Simple**	**Prefixed**	**Suffixed**	**Simple**	**Prefixed**	**Suffixed**
Types of errors	−			−			−		
Affix errors	−	23	10	−	11	11	−	25	4
(omissions; substitutions; neologisms)	−	(11; 6; 6)	(5; 5; 0)	−	(5; 6; 0)	(7; 3; 1)	−	(20; 5; 0)	(1; 2; 1)
Errors on stems	−	14	14	−	17	7	−	9	3
(substitutions; neologisms)	−	(8; 6)	(3;11)	−	(10; 7)	(4; 3)	−	(7; 2)	(2;1)
Substitutions with complex words	3	5	4	8	9	9	5	4	3
Substitutions with simple words	4	0	1	11	3	5	19	13	14
Neologisms	21	7	13	18	10	5	5	5	7
Omissions	10	17	17	15	3	18	38	44	50
Morpheme insertions	5	2	9	12	13	3	0	2	0
Letter deletions in verb endings	1	0	0	0	0	0	3	0	0
Total number of errors	44/119	68/119	68/116	64/119	66/117	58/119	70/117	102/117	81/116
	37%	57%	59%	54%	56%	49%	60%	87%	70%

*Number of errors out of total number of trials for each participant and condition. The table does not report the cases in which insertions of morphemes occurred in addition to other errors: WE inserted additional morphemes to the end of words in two cases of prefix omissions, four cases of prefix substitutions, and one case of stem substitution. SA inserted morphemes to the end of words in three cases of prefix omissions.*

NN produced more errors with both prefixed and suffixed words than with simple words; error rates were similar for prefixed and suffixed words. The effect of Condition on error rates was significant for both the comparison between prefixed and simple words (β = 0.9806, SE = 0.3412, *z* = 2.874, *p* = 0.0041) and that between suffixed and simple words (β = 1.0513, SE = 0.3457, *z* = 3.041, *p* = 0.0024). None of the covariates we tested for inclusion significantly improved the model fit. As for the types of errors produced, there were more affix errors with prefixed words than with suffixed words. The model testing the number of affix errors produced in the two types of derived words revealed a significant effect of Condition (β = −1.0943, SE = 0.4990, *z* = −2.193, *p* = 0.0283), confirming the difference between prefixed and suffixed words on the number of affix errors, as well as a significant effect of Stem Length (β = 0.5221, SE = 0.2374, *z* = 2.199, *p* = 0.0279) suggesting that affix errors additionally increased with increasing length of the stem. Affix errors with prefixed words were more often omissions than substitutions or neologisms. Errors on stems occurred in similar rates for prefixed and suffixed words. The best fit model testing errors on stems showed no evidence for a difference between the two conditions (β = 0.0456, SE = 0.5348, *z* = 0.085, *p* = 0.932). For this model, the model only including the effect of Condition without covariates did not converge; we thus fitted other models, additionally including each of the covariates, and took the model with the best fit. The best-fit model included the covariate Number of Neighbors, which showed no significant effect (β = −0.4011, SE = 0.3247, *z* = −1.235, *p* = 0.217).

As for LG, there was clearly no effect of Condition on his error rates, which were similar for simple, prefixed, and suffixed items (prefixed vs. simple: β = −0.2325 SE = 0.6447, *z* = −0.361, *p* = 0.7184; suffixed vs simple: β = −0.4758, SE = 0.6305, *z* = −0.755, *p* = 0.4504). Instead, the model revealed significant effects of Imageability (β = −0.7838, SE = 0.2705, *z* = −2.897, *p* = 0.0038), Word-Form Frequency (β = −2.3114, SE = 0.6696, *z* = −3.452, *p* = 0.0006), Lemma Frequency (β = 1.8336, SE = 0.6543, *z* = 2.802, *p* = 0.0051), and Session (β = −0.3298, SE = 0.1554, *z* = −2.122, *p* = 0.0338). This suggests that errors increased with increasing lemma frequency, while they decreased with increasing word-form frequency and imageability, and that LG produced fewer errors in the second session. As for affix errors, they were produced in similar amounts for prefixed and suffixed words. The best-fit model showed that the effect of Condition (suffixed vs prefixed) was not significant (β = −0.0195, SE = 1.3757, *z* = −0.014, *p* = 0.9887), while there was a marginal effect of Trial Number (fewer affix errors with increasing trial number; β = −0.8669, SE = 0.4587, *z* = −1.890, *p* = 0.0588). Despite a numerical tendency for more errors on stems with prefixed than suffixed words, the model produced no evidence for a difference between the two conditions (β = −1.1813, SE = 1.3736, *z* = −0.860, *p* = 0.3898), and no other factor significantly improved the model fit.

Finally, SA produced more errors with prefixed words, followed by suffixed and simple items. The larger number of errors with prefixed than simple words turned out to be significant (β = 1.4584, SE = 0.3984, *z* = 3.660, *p* = 0.0003), while there was no significant difference between suffixed and simple items (β = 0.2999, SE = 0.3340, *z* = 0.898, *p* = 0.3693). The best fit model included significant effects of Imageability and Trial Number (respectively: β = −0.5629, SE = 0.1553, *z* = −3.624, *p* = 0.0003; β = 0.3547, SE = 0.1424, *z* = 2.490, *p* = 0.0128), signaling that error rates additionally decreased with increasing imageability and increased as the experiment proceeded. Because, for SA, we observed a larger number of errors with prefixed than suffixed words, we additionally changed the baseline of the factor Condition to “prefixed” in order to directly compare the two types of derived words. The effect of Condition for the comparison between prefixed and suffixed items was significant (β = −1.1585, SE = 0.3979, *z* = −2.911, *p* = 0.0036). SA produced significantly more affix errors with prefixed words than with suffixed words (β = −2.0554, SE = 0.5806, *z* = −3.540, *p* = 0.0004), and affix errors on prefixes were mostly omissions. Finally, there was no evidence for a difference between prefixed and suffixed words in terms of number errors on stems (β = −1.1029, SE = 1.6361, *z* = −0.674, *p* = 0.5002). Neither of the two latter models included additional significant covariates.

The prefixed and suffixed items in our experiment contained a range of different affixes with different meanings or functions. As observed by De Bleser and Bayer (1990), this may have a relevant impact on errors produced on affixes. With exploratory purposes, we built a subset of semantically homogenous items to check whether, once the meaning of the affix is additionally controlled for, the results on affix errors would numerically be in line with those found for the entire item set. This subset included 20 prefixed words containing the negative prefix *un*- (7 adjectives, 13 nouns) and 20 suffixed words containing the negative suffix -*los* (all adjectives), all tested twice. In line with the results we report for the whole set of items, SA and, to some extent, NN produced numerically more affix errors with prefixed than with suffixed words (NN: prefixed 3/40, 8% and suffixed 1/40, 2%; SA: prefixed 9/38, 24% and suffixed 2/38, 5%; LG: no affix errors).

## Discussion

In the present study, we tested three individuals with agrammatic aphasia who, in a preliminary task, showed an impairment for reading aloud morphologically complex words. Our experiment involved reading aloud of simple, prefixed, and suffixed words. We focused on whether prefixed words were more impaired than suffixed words in terms of error rates, as well as on the likelihood of producing errors involving affixes and errors involving stems.

One participant, LG, showed results that are not in line with a profile of morphological impairment. Despite the large number of errors with complex words that LG produced in the preliminary assessments, in the experimental task he produced a similar amount of errors in all conditions. Therefore, for LG, there is no evidence that complex words are more impaired than simple words. There are two possible explanations for why we did not observe an effect in the expected direction. In order to create sets of prefixed, suffixed, and simple items that were comparable for length, we included more non-Germanic words in the simple condition than in the prefixed and suffixed item sets. These words, despite being ordinary words of German, may contain infrequent sound clusters and do not take the standard stress pattern of German, which may make them harder to produce for speakers with language impairment. Hence, the lack of difference between complex words (prefixed and suffixed) and simple items may be due to increased error rates in the simple condition. Indeed, nearly half of the incorrect responses LG produced with simple words (31/64) involved non-Germanic items. Another possible explanation is that many of the items in the preliminary task contained inflectional suffixes, while the main experiment focused on derived words. This may indicate that LG is impaired for reading inflected words, but not derived words. The fact that LG produced a relatively small number of errors selectively affecting the affix, for both prefixed and suffixed words, also speaks against a morphological impairment, at least for derived words.

NN, instead, showed the typical profile of a morphological impairment, with a significant disadvantage for both prefixed and suffixed words as compared to simple words, and similar error rates with the two types of derived words. This is in line with previous studies testing both prefixed and suffixed words (e.g., Job and Sartori, 1984; Kay, 1988; Hamilton and Coslett, 2008), which, however, did not specifically test for differences between these two types of derivations. When we analyzed the types of errors produced with prefixed and suffixed words, we found that prefixes were more likely to be specifically impaired than suffixes. Instead, the number of errors affecting stems were similar in the two conditions.

Finally, SA had yet a different profile. Like NN, she produced significantly more errors with prefixed words than with simple words. In contrast, error rates with suffixed words did not differ from error rates with simple words. Instead, she produced significantly more errors with prefixed words than with suffixed words, suggesting that prefixed words were selectively impaired. Like NN, she also produced significantly more affix errors on prefixes than on suffixes. Errors on stems did not differ in the two conditions.

Let us now come to what we can specifically conclude about prefixed words. When we analyzed error rates, we found that both NN and LG produced similar numbers of errors with prefixed and suffixed words. Instead, for SA, we reported a selective impairment for prefixed words. This dissociation between prefixed and suffixed words can only be explained by positing that, at some level of processing, the relative position of affix and stem influences how prefixed and suffixed words are processed and retrieved. This is in line with previous results from psycholinguistic studies that have reported larger processing costs for prefixed as compared to suffixed words, which the authors explained in terms of more effortful access to the stem when this is in word-final position (Bergman et al., 1988; Colé et al., 1989; Beauvillain, 1996; Ferrari Bridgers and Kacinik, 2017). We therefore suggest that this can also extend to the retrieval of prefixed words in morphological impairment. Retrieving prefixed words would be more costly than retrieving suffixed words, causing larger error rates with prefixed words than with suffixed words. Note that SA seemed to be the participant with the most severe impairment, as reflected by the much larger number of errors she produced compared to the other participants. This may indicate that clear dissociations between prefixed and suffixed words can only be observed in cases of rather severe impairments.

Difficulty in accessing the stem of prefixed words, as compared to suffixed words, may also result in more errors on stems in the prefixed than in the suffixed condition. However, we reported similar numbers of errors on stems in prefixed and suffixed words for all PwAs. It is important to point out that in the present study, unlike in the study by Semenza et al. (2002), morphologically based errors produced with prefixed words involved affixes more often than stems (at least in NN and SA), which is in line with previous evidence on suffixed words (e.g., Rastle et al., 2006). The fact that morphological impairments of derived words generally affect affixes rather than stems implies that the numbers of errors involving stems that we can analyze is relatively small, making it difficult to detect differences between conditions. Future studies may try to address this issue by testing participants whose impairment affects more strongly stems than affixes (such as those reported by Semenza et al., 1990; Semenza et al., 2002).

**FIGURE 1:**
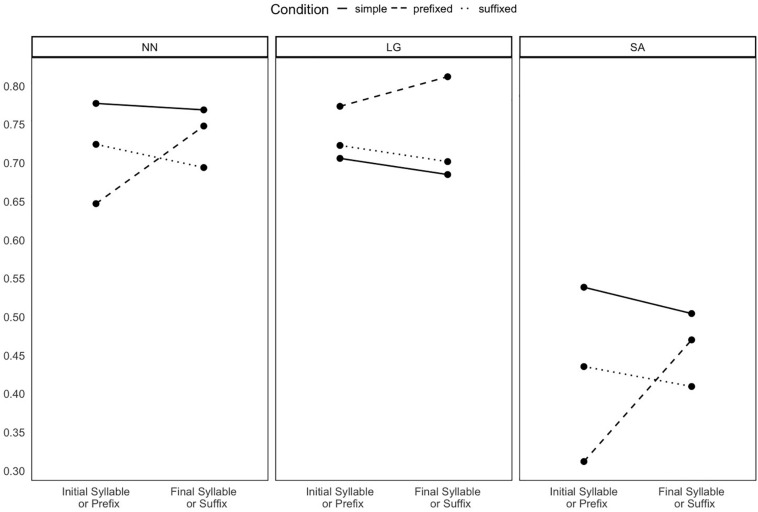
Mean accuracy in the production of initial syllables (or prefixes) and final syllables (or suffixes) in the three conditions, for each PwA across all trials. One point was assigned if the syllable (or affix) was produced correctly, 0.5 if the produced syllable (or affix) preserved at least one letter from the original syllable (or affix), and 0 if the produced syllable (or affix) was entirely different from the original one.

When we analyzed the likelihood of producing affix errors, we found that NN and SA produced more affix errors with prefixes than with suffixes. Prefix errors in both NN and SA were mostly omissions. Figure 1 provides a graphical representation of the proportions of initial word segments (syllables or prefixes) and final word segments (syllables or suffixes) that were preserved. This should provide an idea about whether affix errors only result from an overall pattern of distortions of word beginnings or endings across all conditions. Importantly, the plot suggests that prefix errors in NN and SA cannot be explained by a general tendency of neglecting initial word segments.

The larger likelihood of producing errors that affect prefixes compared to suffixes can be interpreted in terms of the different functions that prefixes and suffixes have in derived words, as discussed in some linguistic literature (e.g., Williams, 1981; Kastovsky, 2005): while prefixes generally do not express the grammatical properties of the derived word (i.e., they are not heads), suffixes generally do. We suggest that, in line with what we predicted, this makes prefixes generally more error-prone than suffixes. A question that remains open is whether the effect we report applies to prefixed words overall, or it is a bare headedness effect (see the literature on compounds, e.g., Jarema et al., 2010; Semenza et al., 2011; Marelli et al., 2013). Future research may address this by directly comparing prefixed words containing head prefixes to the more common case of prefixed words in which the head is the stem. Access to head information may also be assessed by testing production of grammatical gender. This would clarify to what extent headedness plays a role in affix errors even in cases, like LG, for which the number of affix errors fails to reveal differences between prefixes and suffixes. A *post-hoc* descriptive analysis on a sub-set of semantically homogenous items seems to suggest that the effect we report would persist even when controlling for the semantic content of prefixes and suffixes. Further research may include larger, semantically homogeneous sub-sets of items, to test whether the numbers and types of affix errors with prefixes and suffixes vary depending on their meaning or function.

Finally, in line with previous research on morphological impairments (e.g., Funnell, 1987; Rastle et al., 2006), error rates and number of affix errors were, additionally, partly modulated by the stimuli characteristics. For LG and SA, errors decreased with increasing imageability. Additionally, in the case of LG, error rates also decreased with increasing word-form frequency; instead, increasing lemma frequency caused more errors, possibly because of larger error rates with verbs, which have larger lemma frequency. Finally, NN’s rates of affix errors increased for words with longer stems. This highlights once again the importance of controlling for these variables when investigating morphological impairments, both by matching the items across conditions and by including the stimuli properties in the statistical models, so that the model outputs provide the effect of the experimental manipulation (in this case simple, prefixed, and suffixed words) controlled for any other relevant factor.

## Conclusion

The present study investigated the errors produced with prefixed and suffixed words in three individuals with agrammatic aphasia. We report differences between prefixed and suffixed words with regard to error rates in one participant (SA) and with regard to affix errors in two participants (NN and SA). The selective impairment for prefixed words we report for SA makes the present study the first reporting a dissociation between prefixed and suffixed derived words, which had been, until now, never investigated in the literature on morphological impairments. This dissociation can only be accounted for by postulating processing differences between prefixed and suffixed words. In line with previous psycholinguistic studies, we claim that the word-final position of the stem in prefixed words makes their retrieval more costly, and thus prefixed words more difficult to retrieve than suffixed words. Furthermore, in two of the participants (NN and SA) we reported a difference between prefixes and suffixes concerning the likelihood of producing affix errors, with prefixes being more impaired than suffixes. We explained this difference in terms of the different functions of prefixes and suffixes.

Because derivation by prefixation in German is much more widespread than in other Indo-European languages (Smolka et al., 2014; Günther et al., 2019), we believe that our results do not have to do with relative frequency of use of derivational prefixes and suffixes in a specific language, but rather with universal aspects of how prefixed and suffixed words are processed. A question that remains open is at which specific stage of the reading-aloud processes differences between prefixed and suffixed words become relevant. Future research may shed light on this aspect by assessing, in more thorough preliminary testing of each participant, the specific locus of their morphological impairment.

## Data Availability Statement

The datasets generated for this study are available on request to the corresponding author.

## Ethics Statement

The studies involving human participants were reviewed and approved by Ethics Committee of the University of Potsdam (application number 32/2016). The patients/participants provided their written informed consent to participate in this study.

## Author Contributions

LC contributed conception and design of the study, collected the data, performed the statistical analyses, and drafted the manuscript. FB and CS contributed conception and design of the study and revised the manuscript critically for important intellectual content. All authors approved the final version of the manuscript for submission.

## Conflict of Interest

The authors declare that the research was conducted in the absence of any commercial or financial relationships that could be construed as a potential conflict of interest.

## Footnotes

1 Note, however, that performance in the visual and auditory lexical decision tasks, despite being well above chance, was overall relatively poor as compared to what would be expected from the corresponding sub-tests on written and auditory word comprehension in the AAT (at least for NN, and, to some extent, LG). This may be due to the fact that the items used in the task were particularly complex, especially because many of them contained inflectional affixes.

## Appendix: Experimental Items

**TABLE A1:** Simple items.

**Item**	**Class**	**English equivalent**	**Item**	**Class**	**English equivalent**
anonym	Adj	anonymous	locker	Adj	relaxed
Aprikose	N	apricot	löschen	V	clear
arrogant	Adj	arrogant	Marmelade	N	jam
bitter	Adj	bitter	Mission	N	mission
bizarr	Adj	bizarre	munter	Adj	lively
brennen	V	burn	passiv	Adj	passive
dezent	Adj	discreet	perfekt	Adj	perfect
düster	Adj	gloomy	Pfeffer	N	pepper
elegant	Adj	elegant	Pflanze	N	plant
flechten	V	weave	Pflaume	N	plum
fliehen	V	flee	prahlen	V	brag
fließen	V	flow	rauschen	V	sough
forschen	V	research	Rebell	N	rebel
gleiten	V	slide	Referendum	N	referendum
glimmen	V	glow	Region	N	region
grotesk	Adj	grotesque	Restaurant	N	restaurant
heiser	Adj	hoarse	Rezept	N	recipe
heiter	Adj	bright	rutschen	V	slip
immens	Adj	enormous	schalten	V	switch
intakt	Adj	intact	Schenkel	N	leg
Joghurt	N	yogurt	Schinken	N	ham
kaputt	Adj	broken	schmecken	V	taste
klappen	V	fold	Schokolade	N	chocolate
Kloster	N	monastery	schrumpfen	V	shrink
Kognak	N	cognac	schwinden	V	dwindle
Kolonie	N	colony	schwören	V	swear
Konvent	N	convention	simpel	Adj	simple
kreischen	V	screech	spontan	Adj	spontaneous
lauschen	V	listen	trocken	Adj	dry
leugnen	V	deny	Zwetsche	N	plum

**TABLE A2:** Prefixed items.

**Item**	**Class**	**English equivalent**	**Item**	**Class**	**English equivalent**
Desillusion	N	disillusion	unklar	Adj	unclear
Desinteresse	N	lack of interest	unklug	Adj	unwise
Disharmonie	N	disharmony	Unkraut	N	weed
entblößen	V	uncover	Unlust	N	listlessness
entfremden	V	alienate	Unmensch	N	brute
entgleisen	V	be derailed	unmodern	Adj	olde-fashioned
enthaupten	V	behead	unrecht	Adj	wrong
entkräften	V	weaken	unreif	Adj	immature
entladen	V	unload	unsanft	Adj	rough
entspannen	V	relax	unsauber	Adj	unclean
erblinden	V	go blind	unschön	Adj	ugly
erdenken	V	think up	Unschuld	N	innocence
erfinden	V	invent	unseriös	Adj	untrustworthy
erheitern	V	amuse	Unwetter	N	storm
erhitzen	V	heat	unwichtig	Adj	unimportant
Erzbischof	N	archbishop	Urenkel	N	great-grandson
Erzengel	N	archangel	Urkraft	N	elemental force
Erzfeind	N	archenemy	Urmensch	N	caveman
illegal	Adj	illegal	Urpflanze	N	primordial plant
inaktiv	Adj	inactive	Urtext	N	original text
indiskret	Adj	indiscreet	Urvolk	N	primitive man
inexakt	Adj	inexact	Urwald	N	jungle
inkomplett	Adj	incomplete	veralten	V	become outdated
instabil	Adj	unstable	verarbeiten	V	process
irreal	Adj	unreal	verarmen	V	become poor
unbequem	Adj	uncomfortable	verbrennen	V	burn
unfertig	Adj	unfinished	verlernen	V	unlearn
Unfrieden	N	discord	verpflichten	V	commit
ungleich	Adj	unequal	verschlafen	V	sleep through
Unglück	N	bad luck	verspäten	V	delay

**TABLE A3:** Suffixed items.

**Item**	**Class**	**English equivalent**	**Item**	**Class**	**English equivalent**
analysieren	V	analyze	Musiker	N	musician
Apotheker	N	pharmacist	nächtigen	V	spend the night
Autorschaft	N	authorship	namenlos	Adj	nameless
Bruderschaft	N	brotherhood	parteilos	Adj	independent
drahtlos	Adj	wireless	Pensionär	N	pensioner
elternlos	Adj	parentless	Pförtner	N	usher
Erbschaft	N	heritage	präzisieren	V	clarify
farblos	Adj	colorless	probieren	V	try
Feindschaft	N	enmity	problemlos	Adj	unproblematic
festigen	V	consolidate	protestieren	V	protest
folgern	V	conclude	respektieren	V	respect
fristlos	Adj	without notice	respektlos	Adj	disrespectful
Gärtner	N	gardener	sänftigen	V	soften
Glöckner	N	bell ringer	sättigen	V	saturate
gnadenlos	Adj	merciless	schädigen	V	damage
gottlos	Adj	godless	schamlos	Adj	shameless
heimatlos	Adj	homeless	schildern	V	describe
Herrschaft	N	control	schlittern	V	slide
huldigen	V	pay homage to	skrupellos	Adj	unscrupulous
interessieren	V	interest	Sportler	N	athlete
kopieren	V	copy	Städter	N	townsman
kraftlos	Adj	powerless	stimmlos	Adj	unvoiced
kreuzigen	V	crucify	telefonieren	V	phone
Kundschaft	N	clientele	Töpfer	N	potter
legitimieren	V	legitimize	tonlos	Adj	toneless
machtlos	Adj	powerless	Vaterschaft	N	fatherhood
Mediziner	N	doctor	wertlos	Adj	worthless
Milliardär	N	billionaire	ziellos	Adj	aimless
Millionär	N	millionaire	zinslos	Adj	without interest
modernisieren	V	modernize	Zöllner	N	customs officer

**TABLE A4:** List of affixes.

**Affix**	**No. of items**	**Meaning/Function**	**Example**
**Prefixes**			
des-/dis-	3	negation	*Interesse* “interest” *Desinteresse* “indifference”
erz-	3	“arch-”	*Bischof* “bishop” *Erzbischof* “archbischop”
un-	20	negation	*schön* “beautiful” *unschön* “not beautiful”
ur-	7	ancient, original	*Mensch* “human being” *Urmensch* “caveman”
in-/il-/ir-	7	negation	*legal* “legal” *illegal* “illegal”
ent-	7	privative	*laden* “load” *entladen* “unload”
er-	5	resultative	finden “find” erfinden “invent”
ver-	8	privative/resultative	lernen “learn” verlernen “unlearn” alt “old” veralten “become outdated”
**Suffixes**			
-er/-ler/-ner	10	agentive	*Musik* “music” *Musiker* “musician”
-är	3	agentive	*Million* “million” *Millionär* “millionaire”
-schaft	7	collective / description of a status	*Autor* “author” *Autorschaft* “authorship”
-los	20	negation	*kraft* “power” *kraftlos* “powerless”
-ieren	10	action from noun	*Telefon* “phone (N) ” *telefonieren* “phone (V)”
-igen	7	resultative	*fest* “firm” *festigen* “consolidate”
-ern	3	action from noun	*Schild* “sign” *schildern* “describe/outline”
